# Deep Sequencing-Based Transcriptome Analysis of Chicken Spleen in Response to *Avian Pathogenic Escherichia coli (APEC)* Infection

**DOI:** 10.1371/journal.pone.0041645

**Published:** 2012-07-31

**Authors:** Qinghua Nie, Erin E. Sandford, Xiquan Zhang, Lisa K. Nolan, Susan J. Lamont

**Affiliations:** 1 Department of Animal Genetics, Breeding and Reproduction, College of Animal Science, South China Agricultural University, Guangzhou, Guangdong, China; 2 Department of Animal Science, Iowa State University, Ames, Iowa, United States of America; 3 Guangdong Provincial Key Lab of Agro-Animal Genomics and Molecular Breeding and Key Laboratory of Chicken Genetics, Breeding and Reproduction, Ministry of Agriculture, Guangzhou, Guangdong, China; 4 Department of Veterinary Microbiology, College of Veterinary Medicine, Iowa State University, Ames, Iowa, United States of America; Auburn University, United States of America

## Abstract

Avian pathogenic *Escherichia coli* (APEC) leads to economic losses in poultry production and is also a threat to human health. The goal of this study was to characterize the chicken spleen transcriptome and to identify candidate genes for response and resistance to APEC infection using Solexa sequencing. We obtained 14422935, 14104324, and 14954692 Solexa read pairs for non-challenged (NC), challenged-mild pathology (MD), and challenged-severe pathology (SV), respectively. A total of 148197 contigs and 98461 unigenes were assembled, of which 134949 contigs and 91890 unigenes match the chicken genome. In total, 12272 annotated unigenes take part in biological processes (11664), cellular components (11927), and molecular functions (11963). Summing three specific contrasts, 13650 significantly differentially expressed unigenes were found in NC Vs. MD (6844), NC Vs. SV (7764), and MD Vs. SV (2320). Some unigenes (e.g. CD148, CD45 and LCK) were involved in crucial pathways, such as the T cell receptor (TCR) signaling pathway and microbial metabolism in diverse environments. This study facilitates understanding of the genetic architecture of the chicken spleen transcriptome, and has identified candidate genes for host response to APEC infection.

## Introduction

Avian pathogenic *Escherichia coli* (APEC), a gram-negative, facultative anaerobic bacterium, causes intestinal and extra-intestinal infections, septicemia, and mortality in broiler chickens [1]. The most common infectious bacterial disease in poultry, APEC-induced colibacillosis reduces growth and egg production, thereby causing significant economic losses, as well as potentially contaminating poultry products, which generatess a risk for human health [1], [2]. The APEC-O1, O2, O78 serotypes of the O serogroup represent at least half of the total number of isolates [3], [4], and are responsible for over 80% of human septicemia cases world wide [2]. Except for the control of environmental conditions, such as humidity and ventilation, prevention of APEC infection usually relies on antibiotic therapy or vaccine administration. However, vaccines are not fully effective against heterologous APEC strains and there is consumer pressure to reduce the use of antibiotics in food animal production. Elucidating the host resistance mechanisms against APEC infection is a foundational step in developing sustainable strategies to enhance resistance to APEC through development of more effective vaccines and through genetic selection of poultry populations for enhanced innate resistance to APEC.

Until now, the major focus in study of the host:pathogen interaction with APEC has been on the bacteria itself. Some virulence factors or genes responsible for pathogenesis or invasion capacities have been discovered in various APEC strains. With two-dimensional gel electrophoresis, one differentially expressed protein of OmpA was isolated from serum and proposed to be involved in APEC resistance [5]. ExPEC adhesin I has been shown to play a significant role during APEC infection in chickens, as its deletion leads to reduced colonization ability and, moreover, complementation of the adhesin gene restored this ability [6]. By microarray investigation and mutational analysis for confirmation, some upregulated APEC genes have been identified in APEC cultured in APEC-treated chicken serum, and these genes are predicted to contribute to APEC virulence [7]. In addition, some other genes, such as APEC autotransporter adhesin A (*aatA*) and *ibeA* have also been reported to affect APEC infection [8], [9].

**Figure 1:**
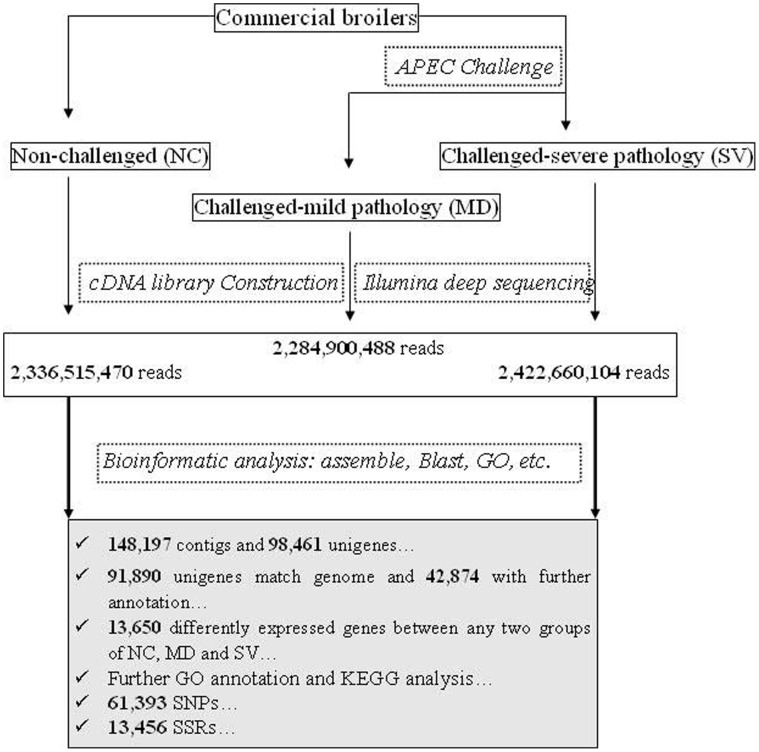
Schematic of Illumina EST analysis. It includes sample preparation, cDNA library construction and Illumina sequencing, data analysis including assemble, blast, GO annotation, gene expression analysis, etc.

Investigations on the host genomic response is also important, so as to reveal the molecular mechanisms of response to APEC infection. With the sequencing of chicken genome [10], the identification of causative genes and markers for APEC susceptibility or resistance at whole genomic or transcriptomic level is practicable and advantageous for genetic selection of poultry with enhanced resistance capabilities. Gene expression profiling by using an avian macrophage microarray revealed 981 differentially expressed chicken ESTs during phagocytosis of *Escherichia coli* (*E. coli*) [11]. A similar study identified 146 common elements modulated by both APEC and *M. synoviae* and exposure to APEC induced higher expression of cytokine genes and genes involved in oxidative burst than *M. synoviae* did [12]. Until now, very few studies at the whole transcriptome level have been reported in response to APEC infection in chicken.

**Table 1:** Summary of draft reads of three libraries by Illumina deep sequencing.

Groups^a^	PE library size (bp)	Read pairs	Read length (bp)	Total residues (bp)
NC	200	14,422,935	81	2,336,515,470
MD	200	14,104,324	81	2,284,900,488
SV	200	14,954,692	81	2,422,660,104
Total	200	43,481,951	81	7,044,076,062

a NC, MD, and SV are three groups of non-challenged, challenged-mild pathology, and challenged-severe pathology, respectively.

Whole transcriptome shotgun sequencing or RNA-seq is an efficient and reliable technology for transcriptomic analysis so as to reveal genetic architecture, to identify sequence variation, and to quantify gene expression [13]. A variety of platforms exist for RNA-Seq, including Illumina Solexa, Roche 454, Life Technology SOLID, and others. Identification of host genetic factors resistance to APEC is of great significance for poultry breeding and production. With use of Illumina deep sequencing of APEC-challenged birds, this study aims to investigate the genetic architecture of the spleen transcriptome, and to discover genes/transcripts and genetic markers for resistance to APEC infection in the chicken.

**Figure 2:**
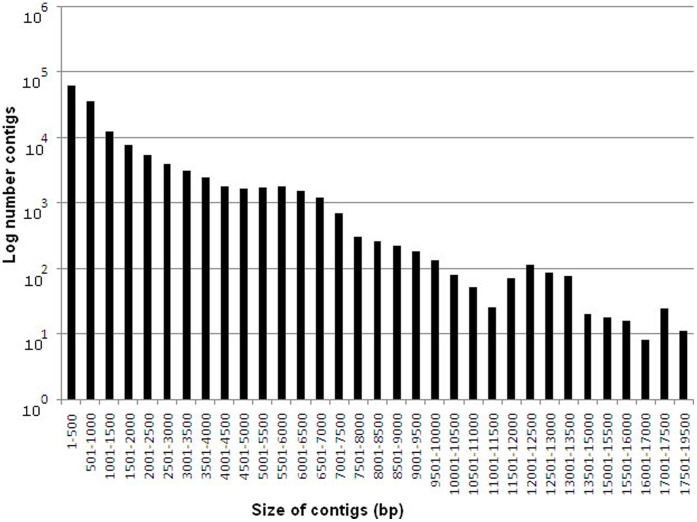
Sequence length distribution of contigs assembled from Illumina reads. All Illumina reads of non-challenged (NC), challenged-mild pathology (MD) and challenged-severe pathology (SV) were used in assembly analysis which gave rise to 148197 contigs. The horizontal and vertical axes show the size of contigs and log number contigs, respectively.

## Results

### Illumina Draft Reads

In this study, spleens of three males were used to prepare one pooled RNA sample for each group of NC, MD and SV. Three cDNA libraries were then constructed to perform Illumina deep sequencing. The schematic of Illumina deep sequencing and analysis are shown in Figure 1. We obtained 14,422,935, 14,104,324, 14,954,692 qualified Illumina read pairs for NC, MD, and SV, giving rise to total residues of 2,336,515,470, 2,284,900,488, and 2,422,660,104 bp, respectively. The overall Illumina read pairs and residues for all samples are 43,481,951 and 7,044,076,062 bp, respectively (Table 1).

**Table 2:** Distribution of contigs and unigenes in chicken genome.

Chromosomes	Counts ofcontigs	Counts ofunigenes	Genomesize^a^
1	20,456	14,336	200,994,015
2	14,092	10,142	154,873,767
3	11,719	8,254	113,657,789
4	11,219	7,407	94,230,402
5	9,245	6,303	62,238,931
6	5,098	3,517	37,400,442
7	5,329	3,534	38,384,769
8	5,071	3,344	30,671,729
9	4,427	2,901	25,554,352
10	4,084	2,777	22,556,432
11	3,204	2,243	21,928,095
12	3,446	2,344	20,536,687
13	3,335	2,268	18,911,934
14	4,016	2,598	15,819,469
15	4,105	2,488	12,968,165
16	222	116	432,983
17	2,996	1,875	11,182,526
18	4,498	1,785	10,925,261
19	3,225	2,066	9,939,723
20	3,204	2,126	13,986,235
21	2,054	1,397	6,959,642
22	834	539	3,936,574
23	1,885	1,257	6,042,217
24	1,363	932	6,400,109
25	547	334	2,031,799
26	1,903	1,197	5,102,438
27	1,516	955	4,841,970
28	1,761	1,164	4,512,026
Z	7,304	5,334	74,602,320
MT	13	10	16,775
Total	142,171	95,543	**1,031,622,801**

a Genome size for each chromosome is based on the released chicken whole genome sequence in either NCBI (ftp://ftp.ncbi.nih.gov/genomes/Gallus_gallus/) or ENSEMBL database (ftp://ftp.ensembl.org/pub/release-63/fasta/gallus_gallus/dna/).

Because some contigs and unigenes have multiple locations in genome, only 134,949 contigs and 91,890 unigenes match with chicken genome.

### Assemble and BLAST Analysis

After assembly analysis based on all Illumina reads, we identified 148,197 contigs with total residues of 195,622,566 bp. The average length of all contigs was 1,320 bp, with the smallest sequence of 300 bp and the largest one of 19,212 bp. The sequence length distribution of contigs is indicated in Figure 2 and Table S1. Analysis of nucleotide content within all contigs showed that the content of A, T, C, G were 26.45% (51,750,884 nucleotides), 26.52% (51,887,566), 23.83% (46,619,027), and 23.19% (45,365,089), respectively, giving rise to an overall GC content of 47.02% in the chicken whole transcriptome.

**Figure 3:**
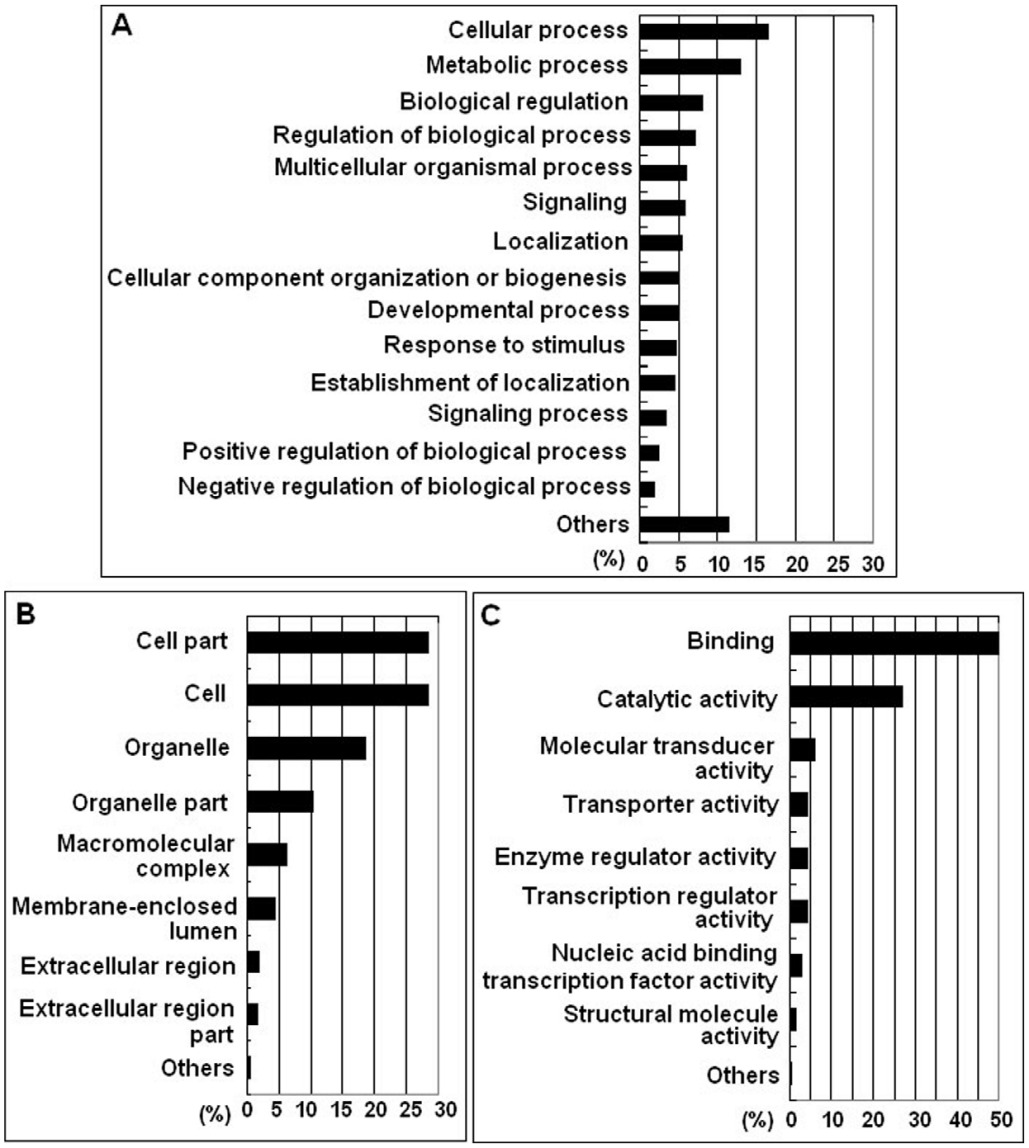
Functional classification of chicken transcriptome. (A) GO: Biological process. (B) Cellular component. (C) GO: Molecular function. Each transcript or gene generally has multiple functions.

**Figure 4:**
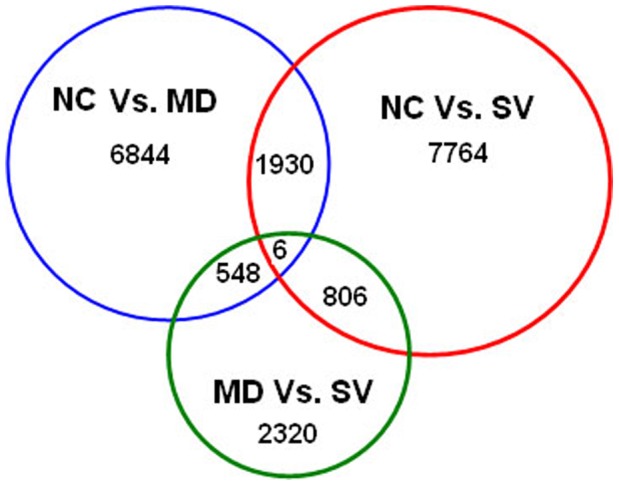
Differentially expressed genes that are unique or shared among three groups of NC, MD and SV. NC Vs. MD refers to the comparison between non-challenged (NC) and challenged-mild pathology (MD) groups. NC Vs. SV refers to the comparison between NC and challenged-severe pathology (SV) groups. MD Vs. SV refers to the comparison between MD and SV groups. Numbers in each section of the figure indicate the numbers of differently expressed genes in the indicatedcomparison.

Further assembly analysis showed that all contigs contributed to 98,461 unigenes. BLAST analysis with the known chicken genome sequence indicated that 134,949 contigs and 91,890 unigenes match the chicken genome. The distributions of contigs and unigenes in chicken chromosomes are described in Table 2.

**Table 3:** Involvement of differentially expressed genes in predicted pathways by KEGG enrichment analysis.

No.	Pathways	#unigenes^1^	upval	dpval	enrichment
1	Metabolic pathways	514 (252)	1.39E−60	1	yes
2	Purine metabolism	418 (199)	1.04E−46	1	yes
3	Thiamine metabolism	359 (181)	9.06E−47	1	yes
4	T cell receptor signaling pathway	105 (38)	1.80E−06	0.999999	yes
5	Biosynthesis of secondary metabolites	98 (53)	2.15E−17	1	yes
6	Lysine degradation	75 (42)	1.48E−13	1	yes
7	Drug metabolism - other enzymes	71 (35)	6.33E−10	1	yes
8	Tropane, piperidine and pyridine alkaloid biosynthesis	56 (30)	8.95E−10	1	yes
9	Microbial metabolism in diverse environments	29 (22)	2.61E−11	1	yes
10	Oxidative phosphorylation	28 (21)	2.32E−11	1	yes
11	Biosynthesis of phenylpropanoids	19 (13)	1.09E−06	1	yes
12	Metabolism of xenobiotics by cytochrome	18 (9)	0.001988018	0.999624	yes
13	Drug metabolism - cytochrome	18 (9)	0.001988018	0.999624	yes
14	Phenylalanine metabolism	17 (14)	4.38E−08	1	yes
15	beta-Alanine metabolism	15 (8)	0.00116723	0.999828	yes
16	Methane metabolism	13 (9)	5.11E−05	0.999996	yes
17	Glycolysis/Gluconeogenesis	12 (10)	2.44E−06	1	yes
18	Phenylpropanoid biosynthesis	12 (9)	2.22E−05	0.999999	yes
19	Histidine metabolism	6 (6)	0.00010714	1	yes
20	Tyrosine metabolism	6 (6)	0.00010714	1	yes

1 Refer to the numbers of involved total unigenes and differently expressed unigenes (in bracket).

### GO Assignments

Among 97,491 assembled unigenes, 12,272 were successfully annotated by GO assignments, belonging to one or more of the three categories: biological process, cellular component, and molecular function. Among the annotated unigenes, 11,664 are involved in various biological process categories, including cellular process (9,876 unigenes; 16.33%), metabolic process (7,808; 12.91%), biological regulation (4,969; 8.22%), regulation of biological process (4,391; 7.26%), and others (Figure 3A). Further, 11,927 unigenes are involved in cellular component categories, including cell part (11,936; 28.38%), cell (11,936; 28.38%), organelle (7,850; 18.66%), organelle part (4,309; 10.25%), macromolecular complex (2,589; 6.16%), membrane-enclosed lumen (1,842; 4.38%), extracellular region (727; 1.73%), extracellular region part (669; 1.59%), and others (200; 0.48%) (Figure 3B). In addition, 11,963 unigenes are involved in molecular function catgories, including binding (10,141; 49.66%), catalytic activity (5,442; 26.65%), molecular transducer activity (1,271; 6.22%), transporter activity (860; 4.21%), enzyme regulator activity (852; 4.17%), transcription regulator activity (823; 4.03%), nucleic acid binding transcription factor activity (598; 2.93%), structural molecule activity (312; 1.53%), and others (120; 0.59%) (Figure 3C).

**Table 4:** Differentially expressed genes involved in the T cell receptor (TCR) signaling pathway.

No.	Gene^1^	Description
1	MAPKSP1	PREDICTED: mitogen-activated protein kinase scaffold protein 1-like isoform 1#PREDICTED: similar to dual adaptor of phosphotyrosine and 3-phosphoinositides#PREDICTED: hypothetical protein#PREDICTED: dual adapter for phosphotyrosine and 3-phosphotyrosine and 3-phosphoinositide-like#PREDICTED: ragulator complex protein LAMTOR3-like
2	PTPN13	PREDICTED: tyrosine-protein phosphatase non-receptor type 13-like
3	PTPRF	PREDICTED: receptor-type tyrosine-protein phosphatase F-like, partial
4	PPP1CC	serine/threonine-protein phosphatase PP1-gamma catalytic subunit#mCG129661, isoform CRA_c
5	BTAF1; Dusp11	PREDICTED: similar to Dual specificity phosphatase 11 (RNA/RNP complex 1-interacting)#PREDICTED: TATA-binding protein-associated factor 172-like
6	PP2C-epsilon	PREDICTED: similar to protein phosphatase 2C epsilon
7	PTPN23	LOC100170608 protein#PREDICTED: tyrosine-protein phosphatase non-receptor type 23-like
8	PTPN14	PREDICTED: similar to protein tyrosine phosphatase, non-receptor type 14 isoform 2#protein tyrosine phosphatase, non-receptor type 14, isoform CRA_c#PREDICTED: similar to protein tyrosine phosphatase, non-receptor type 14 isoform 1
9	SBF1	PREDICTED: similar to tyrosine kinase#myotubularin-related protein 5 isoform 1
10	PTPN11	PREDICTED: tyrosine-protein phosphatase non-receptor type 11-like
11	KIAA0371	PREDICTED: similar to Cysteine rich protein 2#PREDICTED: similar to KIAA0371
12	EYA3	PREDICTED: eyes absent homolog 3-like isoform 2#PREDICTED: similar to eyes absent 3
13	CD148	receptor-type tyrosine-protein phosphatase eta
14	PTPRQ	PREDICTED: phosphotidylinositol phosphatase PTPRQ-like
15	CD45; PTPRC	receptor-type tyrosine-protein phosphatase C
16	p70; STS1	PREDICTED: similar to KIAA1959 protein
17	N/A	PREDICTED: hypothetical protein
18	Dusp22	PREDICTED: similar to RP23-217J3.1
19	Ptprr	PREDICTED: receptor-type tyrosine-protein phosphatase R-like
20	Ppp2r2a	serine/threonine-protein phosphatase 2A 55 kDa regulatory subunit B alpha isoform#hypothetical protein PANDA_002366#PREDICTED: serine/threonine-protein phosphatase 2A 55 kDa regulatory subunit B delta isoform-like#PREDICTED: alpha isoform of regulatory subunit B55, protein phosphatase 2 isoform 1#unnamed protein product#PREDICTED: protein phosphatase PP2A 55 kDa regulatory subunit isoform 1
21	PPM1A	PREDICTED: protein phosphatase 1A (formerly 2C), magnesium-dependent, alpha isoform#PREDICTED: similar to protein phosphatase 2C alpha; PP2Calpha
22	Dusp11	PREDICTED: similar to Dual specificity phosphatase 11 (RNA/RNP complex 1-interacting)#PREDICTED: RNA/RNP complex-1-interacting phosphatase-like
23	PTPRQ	PREDICTED: phosphotidylinositol phosphatase PTPRQ-like
24	PTPN6	tyrosine-protein phosphatase non-receptor type 6
25	PTPN1; PTP1B	tyrosine-protein phosphatase non-receptor type 1#PREDICTED: similar to PDE4DIP protein#protein tyrosine phosphatase#PREDICTED: similar to phosphodiesterase 4D interacting protein#PREDICTED: tyrosine-protein phosphatase non-receptor type 1-like
26	SSH-2	PREDICTED: similar to slingshot-2L#PREDICTED: protein phosphatase Slingshot homolog 2-like
27	PTPN22	PREDICTED: similar to Tyrosine-protein phosphatase non-receptor type 22 (Hematopoietic cell protein-tyrosine phosphatase 70Z-PEP) (Lymphoid phosphatase) (LyP)
28	LCK	LCK_CHICKRecName: Full = Proto-oncogene tyrosine-protein kinase LCK; AltName: Full = Protein-tyrosine kinase C-TKL; AltName: Full = p56tk1
29	CTDNEP1	CTD nuclear envelope phosphatase 1
30	Ppm1k	PREDICTED: similar to Protein phosphatase 1K (PP2C domain containing)#Hyperion protein, 419 kD isoform#PREDICTED: protein phosphatase 1K, mitochondrial-like
31	Ppm1f	PREDICTED: similar to Protein phosphatase 1F (PP2C domain containing)
32	PTPN5; Step	PREDICTED: tyrosine-protein phosphatase non-receptor type 5-like
33	RAD9A;RAD9	cell cycle checkpoint control protein RAD9A#putative protein phosphatase 1 catalytic subunit alpha
34	PTPRB; PTPB	PREDICTED: similar to Protein tyrosine phosphatase, receptor type, B
35	PTPN21	PREDICTED: tyrosine-protein phosphatase non-receptor type 21-like
36	MTMR8	myotubularin-related protein 8
37	SSH1	PREDICTED: similar to Slingshot homolog 1 (Drosophila)
38	PIX1	PREDICTED: similar to partner of PIX 1

1 Genes are named based on NCBI gene database (http://www.ncbi.nlm.nih.gov/gene/). N/A means not available.

**Table 5:** Comparison of gene expression that differs significantly among NC, MD and SV for CD148, CD45 and LCK.

Gene	Expression value^1^			NC vs. MD		NC vs. SV		MD V = vs. SV	
	NC	MD	SV	Fold change^2^	q value^3^	Fold change^2^	q value^3^	Fold change^2^	q value^3^
CD148	955	991	1191	−0.2113	0.0128	−0.4345	1.45E−10	−0.2232	0.0091
CD45	19572	17645	17329	−0.0084	0.7551	0.0597	0.000912	0.0681	0.0006
LCK	1465	1035	837	0.3434	1.63E-07	0.6917	1.67E−27	0.3484	1.76E-05

1 The expression value is based on the obtained reads within the specific gene in the three groups of [non-challenged (NC), challenged-mild pathology (MD), and challenged-severe pathology (SV)].

2 Refers to normalized fold changes.

3 The q-value was calculated according to Benjamini et al. (1995) [38].

**Figure 5:**
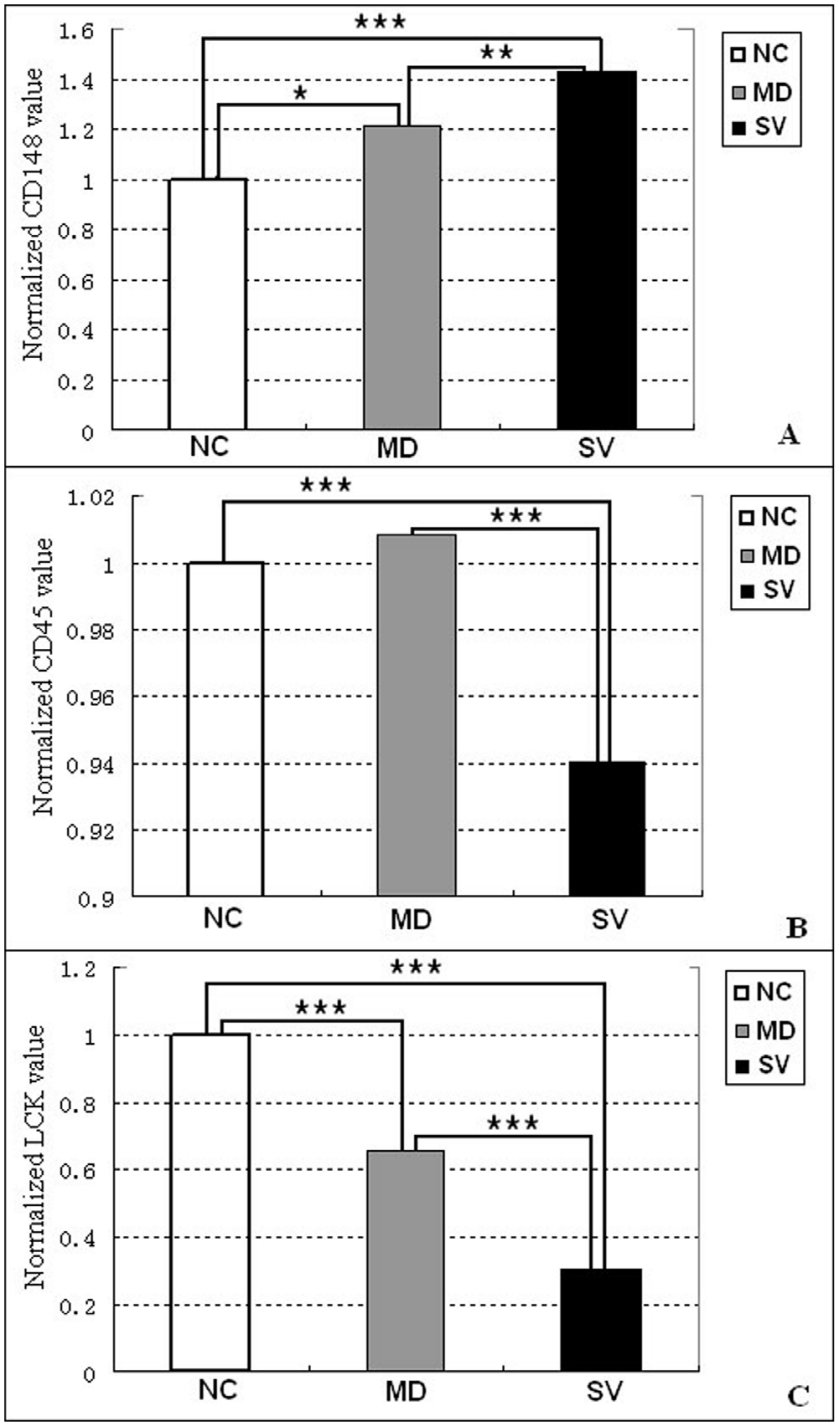
Comparison of CD148, CD45 and LCK expression among NC, MD and SV. (A) CD148. (B) CD45. (C) LCK. NC, MD and SV stand for the groups of non-challenged, challenged-mild pathology, and challenged-severe pathology, respectively. The normalized expression value of NC is set as 1, by which the value of MD and SV are determined. *, ** and *** represent q-value significance at the level of 0.05, 0.01 and 0.001, respectively.

### Differentially Expressed Genes

Comparison of gene expression showed that a total of 13,650 unigenes were differentially expressed between any two-way comparison of the groups of NC, MD and SV (fold changes ≥2 or ≤−2; q value <0.01), including 6,844 significantly expressed isogenes between NC and MD (NC Vs. MD), 7,764 between NC and SV (NC Vs. SV), and 2,320 between MD and SV (MD Vs. SV). Moreover, 1,930 unigenes were significantly differentially expressed in both NC Vs. MD and NC Vs. SV, and 548 in NC Vs. MD and MD Vs. SV, as well as 806 in NC Vs. SV and MD Vs. SV. Only 6 unigenes were significantly differentially expressed in all of NC Vs. MD, NC Vs. SV, and MD Vs. SV. Numbers of all differentially expressed genes among the three groups of NC, MD and SV are illustrated in Figure 4. In addition, there are 531, 115 and 134 unigenes that are uniquely expressed in the group of NC, MD, and SV, respectively.

### Metabolic Pathways by KEGG Analysis

KEGG enrichment analysis showed that the differentially expressed genes were involved in twenty predicted pathways at a significant level. The pathways and involved unigene numbers are metabolic pathways (514 unigenes and 252 differently expressed unigenes), purine metabolism (418; 199), thiamine metabolism (359; 181), T cell receptor signaling pathway (105; 38), biosynthesis of secondary metabolites (98; 53), lysine degradation (75; 42), drug metabolism-other enzymes (71; 35), tropane, piperidine and pyridine alkaloid biosynthesis (56; 30), microbial metabolism in diverse environments (29; 22), oxidative phosphorylation (28; 21), biosynthesis of phenylpropanoids (19; 13), metabolism of xenobiotics by cytochrome (18; 9), drug metabolism-cytochrome (18; 9), phenylalanine metabolism (17; 14), beta-alanine metabolism (15; 8), methane metabolism (13; 9), glycolysis/gluconeogenesis (12; 10), phenylpropanoid biosynthesis (12; 9), histidine metabolism (6; 6), and tyrosine metabolism (6; 6). All pathways and related information are described in Table 3.

A total of 38 differentially expressed genes are involved in the T cell receptor (TCR) signaling pathway, which has important functions in animal immunity (Table 4). Three crucial genes in this pathway, CD148, CD45, and LCK, exhibited significantly different expression among NC, MD and SV groups (Table 5). The CD148 was significantly up-regulated in SV (P<0.001) and MD (P<0.05) compared with NC (Figure 5A); in contrast, LCK was significantly lower in SV (P<0.001) and MD (P<0.001) compared with NC (Figure 5C). The CD45 gene, was expressed at a significantly lower level in SV compared with NC (P<0.001) and with MD (P<0.001), but was not significantly different between NC and MD (P>0.05) (Figure 5B).

## Discussion

### Assembly, Blast and GO Analysis

The high-throughput sequence data obtained by Illumina deep sequencing contributes to the understanding of the genetic architecture of chicken transcriptome. In this study, we pooled RNA from multiple individuals to generate one sample, and subsequently performed Illumina deep sequencing. This pooling strategy was widely used in some similar studies [14], [15]. As a result, we generated 148,197 contigs for 195.6 Mb residues of chicken spleen transcriptome based on 43,481,951 Illumina read pairs. Considering all contigs, the overall GC content of the transcriptome was calculated to be 47.02%, which is very close to that reported for genome-wide exons (i.e. 47.00% for GGA4q), but much higher than that of genome-wide introns (i.e. 40.00% for GGA4q) [10]. We obtained a total of 98,461 unigenes by further assembly analysis, which was more than all predicted genes (20,000–23,000) in chicken genome [10]. We compared our unigenes with NCBI unigene database using blastn, and found that 35,056 unigenes of this study have high similarity with NCBI unigenes (19,218) using 95% identity cutoff. Compared with our unigenes, 1,103 of 19,218 NCBI unigenes are covered by 100% in length, and moreover, 4,326, 2,151 and 2,373 unigenes show the coverage of 90–99%, 80–89%, and 70–79%, respectively. Some unigenes of this study represent for the same NCBI unigene probably because some genes show low expression level in spleen and our Illumina sequences are less deep enough to generate the complete transcript by assemble analysis. Meanwhile, some unigenes of this study are longer than corresponding unigene in NCBI, i.e. 3,661 unigenes were longer than NCBI corresponding unigenes using a cutoff (less than 90% coverage for our unigenes and more than 90% coverage for NCBI unigenes). In addition, there are 7,276 unigenes which couldn’t be mapped to chicken genome. These unmapped unigenes still belong to chicken transcriptome, and some if not all of them might be ncRNA which need to be studied in future. Alternative splicing is also very common in chicken genome, as it was previously estimated that 40−60% of all genes and 74% of multiexon genes are alternatively spliced in the human genome [16]. GO annotation showed that some unigenes were involved in the three categories of biological process (11,664 unigenes), cellular component (11,927), and molecular function (11,963).

The chicken whole genome was sequenced in 2004 [10], but no major update has been published since then. Recently, the genomes of two other avian species, i.e. turkey and zebra finch, were sequenced [17], [18]. The known chicken genome is 1,063 Mb in total length, of which 933 Mb were localized in 29 autosomes (GGA1-28 and 32) and sex chromosomes (Z and W), and the remaining residues were unlocalized [10], [19]. In the current study, blast analysis with the chicken genome showed that most contigs and unigenes mapped to GGA1-5 and the Z chromosome, corresponding to the fact that these macrochromosomes contain a major part of the chicken genome [19]. It is logical that no contig or unigene was found for the known GGA32, because only 1,028 bp of sequences are available in this chromosome (ftp://ftp.ncbi.nih.gov/genomes/Gallus_gallus/).

Even though the chicken transcriptome of various tissues has been investigated by cDNA microarray or gene chip [7], [11], [12], this technology fails to detect sequence variation and to recognize new genes or transcripts. With emergence of second generation sequencing, RNA-seq is a more powerful approach for transcriptome analysis [13]; however, such investigations in chickens and other birds are very limited. Recently, a total of 856,675 Roche 454 reads were obtained in crows and further expression analysis indicated a general pattern of ineffective dosage compensation in that species [20], [21]. The results of the current study as well as 148,197 Illumina reads are useful resource for further investigation on chicken transcriptome.

### Differently Expressed Genes for APEC

Comparison of gene expression among the different treatment groups in the current experiment is helpful for identification of candidate genes underlying response and resistance to APEC infection in chicken. Many fewer differentially expressed unigenes were found in the MD Vs. SV contrast (2,320) compared with that of NC Vs. MD (6,844) and NC Vs. SV (7,764), revealing that there is greater difference between infected and non-infected states than between mild and severe infections. Among the differently expressed genes, there are 531, 115 and 134 unigenes that specifically express in NC, MD, and SV groups, respectively. Our previous study with cDNA microarray technology revealed 1,101 significantly expressed genes between SV and NC [22]. Because RNA-Seq can recognize new unigenes or unique isoforms present in chicken transcriptome, it should be more powerful in expression analysis. Further, KEGG prediction and GO analysis showed that these differently expressed genes were involved in a couple of major pathways. It is notable that 38 and 22 unigenes are contained in the two predicted pathways of TCR signaling pathway and microbial metabolism in diverse environments, respectively, suggesting that these unigenes are candidate genes for APEC infection in chicken.

The TCR signaling in response to antigen recognition can induce integrin to facilitate T-cell activation and thus the TCR signaling pathway has a central role in the adaptive immune response [23], [24]. Within the TCR signaling pathway, CD148, CD45 and LCK are crucial genes that regulate the signal transduction throughout the entire network [25], [26]. It is notable that CD45 and CD148 are specifically required for *L. pneumophila* phagocytosis and effector translocation [27], suggesting that they are involved in the interaction between host and bacterium. It has been proposed that CD45 is alone sufficient for TCR triggering [28] and, moreover, CD45 deficiency results in a severe combined immunodeficiency phenotype [26]. In the current study, both CD45 and LCK were significantly downregulated in SV compared to NC and MD (P<0.001), which supports the critical function of CD45 in the immune response to APEC in chicken. The expression of CD148 was upregulated in SV compared with NC (P<0.001) and MD (P<0.01) in this study. The true function of CD148 in the immune system remains unclear and there are contrary opinions regarding whether CD148 is dispensable for normal growth and development [26]. The identified unigenes in the TCR signaling pathway are candidate genes for APEC infection in chicken, and warrant additional functional confirmation by further investigation.

The cytokine interleukin-1 beta (IL1B) is known as a “master” cytokines and plays a great role on the process of anti-infectious protection. In the pathogenesis of APEC, we found that IL1B expression was significantly up-regulated in both SV and MD compared to NC, and moreover SV showed higher IL1B level than MD. It indicated that IL1B was a key cytokine responsible for the inflammation process caused by APEC infection. A similar result was also found in cow mastitis. Investigation on the global transcription of Mild priming primary mammary epithelial cells (MEC) of cow for 12 h with lipopolysaccharide (LPS) (100 ng/ml) before stimulated with heat inactivated E. coli bacteria showed that, the expression of IL1B was significantly down-regulated to inhibit inflammation [29]. Moreover, it was predicted that IL1B could directly regulate 44 differently expressed genes in the process of LPS priming-mediated modulation of the E. coli-elicited response [29].

Toll-like receptors are important factors for immune response. In this study, TLR4 was significantly up-regulated in MD (2.3 folds) and SV (2.9 folds) compared to NC, which was consensus to the study of TLR4 in human. After co-stimulation the T24 human bladder carcinoma cell with E. coli and lactobacilli, TLR4 were significantly increased in both mRNA and protein level, and inhibition of TLR4 blocked the lactobacilli potentiation of NF-kappaB [30]. TLR2 were also up regulated in MD (3.0 folds) and SV (3.3 folds) compared to NC. It was reported the TLR2 subfamily were involved in the avian response to C. perfringens challenge [31].

Compared to NC, the L-phenylalanine oxidase IL4I1 up-regulated its expression in SV and MD at 20 and 39 folds respectively. Meanwhile, IL4I1 expression in SV was about 2 fold higher than MD. It indicated that IL4I1 facilitates the pathogens of APEC. In human, it was proved that IL4I1 improve tumor growth by inhibiting the CD8(+) antitumor T-cell response [32].

## Materials and Methods

### Ethics Statement

The APEC challenge experiment and sample collection were approved by the Iowa State University Institutional Animal Care and Use Committee (# 11-07-6460-G).

### APEC Challenge Experiment and Sample Preparation

A total of 240 non-vaccinated commercial male broilers at 4 weeks age were challenged with 0.1 ml APEC O1 (10E8 colony forming units) by the intra-air sac route into the left thoracic airsac. Another 120 non-vaccinated males were non-challenged (NC) but treated with 0.1 ml phosphate buffered saline (PBS). All detailed information on the APEC O1 strain and challenge design and procedures was previously described [22]. Birds were euthanized and necropsy was performed at one day post challenge. Based upon pathological finds a summarized lesion score, ranging from 0 to 7, was determined for each bird. Birds with lesion scores of 0–2 were regarded as mild pathology (resistant phenotype), and those scoring 4–7 as severe pathology (susceptible phenotype). Subsequently, spleens from three groups [non-challenged NC, challenged-mild pathology (MD) and challenged-severe pathology (SV)] were subjected to Illumina deep sequencing to investigate the dynamic responses of chicken transcriptome. The recorded lesions (mean ± standard deviation) for NC, MD, and SV groups were 0.00±0.00, 0.50±0.58, and 5.25±1.26, respectively.

### RNA Isolation, cDNA Library Construction and Illumina Deep Sequencing

For each group, three spleens were randomly chosen and shipped on RNAlater (Applied Biosystems) to Shanghai Majorbio Bio-pharm Biotechnology Co., Ltd. (Shanghai, China), where total RNA was isolated from each spleen by trizol (Invitrogen, CA, USA). Then, samples of three individuals were pooled within each group in equal amounts to generate one mixed sample per group by RNA pooling. These three mixed RNA samples were subsequently used in cDNA library construction and Illumina deep sequencing.

Three cDNA libraries were prepared using the TruseqTM RNA sample prep Kit (Illumina, San Diego, CA USA) following the manufacturer’s instructions. First, magnetic beads containing poly-T molecules were used to purify mRNA from 10 µg of total RNA. Second, the three samples were chemically fragmented and reverse transcribed into cDNA. Third, end repair and A-base tailing was performed and then Illumina adapters were ligated to the cDNA fragments.

After a gel size fractionation step to extract fragments of 300 bp, 29 µL of the purified samples were amplified by 15-cycle PCR. Amplified products were validated and quantified using an Agilent 2100 bioanalyzer and the DNA 1000 Nano Chip Kit (Agilent, Technologies, Santa Clara, CA, USA). Libraries were loaded onto the channels of the flow cell at 8 pM concentration. Sequencing was performed on the Genome Analyzer IIx (Illumina, San Diego, CA, USA) by running 81+7+81 cycles using Illumina’s cBot Paired End Cluster Plate Kit and 36 Cycle Sequencing Kit according to the manufacturer’s instructions.

### Bioinformatic Analysis

#### Reads trimming and assembly

For each of the sequencing reads, low quality bases (Sanger base quality <20) of 3′ ends were first trimmed using in-house perl scripts and then the sequencing adapters were trimmed using fastx_toolkit software (http://hannonlab.cshl.edu/fastx_toolkit/). All Illumina reads of three samples (NC, MD and SV) were assembled by Trinity software using default parameters [33].

### Transcriptome Annotation

The isogenes were compared with the protein nonredundant database using BlastX with E values less than 1.0×10^−5^ (E values less than 1.0×10^−5^ were considered as significant) [34]. Gene ontology (GO) terms were extracted from the best hits obtained from the BlastX against the nr database (E value ≤1.0×10^−6^) using blast2go, and then sorted for the GO categories using in-house perl scripts [35]. Metabolic pathway analysis was performed using the Kyoto Encyclopedia of Genes and Genomes (KEGG).

### Expression Analysis

Reads from each samples were mapped to isogenes using bowtie software respectively using corresponding parameters for pair ends reads and single reads [36]. The expression of each gene was calculated using the numbers of reads mapping to its isogenes. For calculating gene expression correctly, reads which aligned to different isogenes of the same gene were only counted once. Reads which have best alignments to more than one gene were not counted. Both reads from a read pair were removed if one read aligned to one gene and the other aligned to another gene. The differentially expressed genes were analyzed using the R package DEGseq and the Benjamini q-value was calculated [37], [38]. Gene Ontology and KEGG pathway enrichment analysis was performed by using the GOseq package, which took gene length bias into account, using a 0.05 cutoff for the false discovery rate [39].

The according to et al. (1995).

## Supporting Information

Table S1 **Sequence length distribution of 148,197 assembled contigs.** Frequencies refer to the percentage of the contigs with each different sequence length.
(DOC)
