# Supplementary material for: Deep Sequencing-Based Transcriptome Analysis of Chicken Spleen in Response to Avian Pathogenic Escherichia coli (APEC) Infection
Source: PLoS One. 2012 Jul 31;7(7):e41645. doi: 10.1371/journal.pone.0041645 (PMC3409229; doi:10.1371/journal.pone.0041645)
Supplement: Table S1 — Sequence length distribution of 148,197 assembled contigs. Frequencies refer to the percentage of the contigs with each different sequence length. (DOC) [file pone.0041645.s001.doc]

**Table S1 Sequence length distribution of 148,197 assembled contigs.**

| Length (bp) | Counts | Percentage |
| --- | --- | --- |
| 1-500 | 64,247 | 43.35 |
| 501-1000 | 36,286 | 24.48 |
| 1001-1500 | 12,738 | 8.60 |
| 1501-2000 | 7,692 | 5.19 |
| 2001-2500 | 5,406 | 3.65 |
| 2501-3000 | 4,058 | 2.74 |
| 3001-3500 | 3,160 | 2.13 |
| 3501-4000 | 2,439 | 1.65 |
| 4001-4500 | 1,838 | 1.24 |
| 4501-5000 | 1,652 | 1.11 |
| 5001-5500 | 1,718 | 1.16 |
| 5501-6000 | 1,798 | 1.21 |
| 6001-6500 | 1,558 | 1.05 |
| 6501-7000 | 1,211 | 0.82 |
| 7001-7500 | 692 | 0.47 |
| 7501-8000 | 307 | 0.21 |
| 8001-8500 | 257 | 0.17 |
| 8501-9000 | 223 | 0.15 |
| 9001-9500 | 183 | 0.12 |
| 9501-10000 | 134 | 0.09 |
| 10001-10500 | 80 | 0.05 |
| 10501-11000 | 52 | 0.04 |
| 11001-11500 | 25 | 0.02 |
| 11501-12000 | 72 | 0.05 |
| 12001-12500 | 113 | 0.08 |
| 12501-13000 | 85 | 0.06 |
| 13001-13500 | 76 | 0.05 |
| 13501-15000 | 20 | 0.01 |
| 15001-15500 | 18 | 0.01 |
| 15501-16000 | 16 | 0.01 |
| 16001-17000 | 8 | 0.01 |
| 17001-17500 | 24 | 0.02 |
| 17501-19500 | 11 | 0.01 |
| Total | 148,197 | 100 |

Frequencies refer to the percentage of the contigs with each different sequence length.
